# Detecting Clusters of MRSA

**DOI:** 10.1371/journal.pmed.0030084

**Published:** 2006-01-10

The incidence of methicillin-resistant *Staphylococcus aureus* (MRSA) in hospitals continues to rise globally. *S. aureus* infections, including MRSA, occur most frequently in patients with weakened immune systems in hospitals and healthcare facilities, such as nursing homes and dialysis centers. Healthcare-associated *S. aureus* infections include surgical-wound infections, bloodstream infections, and pneumonia. Factors associated with the spread of MRSA infections in hospitals include skin-to-skin contact, wounds, contaminated items and surfaces, and poor hygiene. Control measures have focused on hand hygiene, restriction of antibiotics, and the detection and isolation of colonized and infected patients.

Strains resistant to methicillin are a major concern in hospitals because of the high mortality rate associated with infections caused by them and the stringent hygienic requirements needed for patients who carry MRSA. Experts say the focus of most national guidelines is the detection and isolation of colonized and infected patients. However, developing rapid detection techniques has not been easy.

Outbreaks are usually identified from laboratory test results and patients' charts—a time-consuming procedure. In established outbreaks, molecular typing (e.g., pulsed-field gel electrophoresis [PFGE]) is usually used to track the outbreak. To improve the speed of typing, DNA sequence-based approaches, such as multi-locus sequence typing (MLST), are being used but are still too expensive for routine use and have lower discriminatory power compared with PFGE.

Frenay et al. became the first group to use a single-locus sequence typing method for *S. aureus*, by using the sequence of the polymorphic region X of the *S. aureus* protein A gene *(spa)* for typing. However, the full potential of this method has not been exploited thus far. Recently, however, a software program was developed so that the *spa* sequences could be analyzed automatically and linked to a database integrated with epidemiological information.

In this month's *PLoS Medicine*, Alexander Mellmann, Dag Harmsen, and colleagues compared this approach with classical surveillance techniques (frequency, and infection control professional [ICP] alerts) to investigate whether an automated system could complement or replace the labor-intensive traditional methods. The study, performed at a German tertiary hospital from 1998–2003, found that only five of the 13 “true” clusters were detected as clusters by visual screening of laboratory reports by the ICP. By contrast, clonal alerts ( *spa* typing and epidemiological data) were more sensitive than the classical techniques.

The authors suggest that *spa* typing and automated alerts could offer a useful early-warning system, which could also be used to model infection dynamics and estimate important epidemiological parameters. The combination of medical informatics and molecular laboratory techniques could help clinicians prevent limited clusters of preventable MRSA from expanding into large-scale outbreaks. Laboratory-based surveillance has another advantage: clusters occurring throughout the hospital could be identified at a single, central data-collection point.

One limitation of clonal alerts and classical techniques, however, is that they all rely on pre-defined rules, which means that unusual patterns of outbreaks might go undetected. A way to get around this limitation might be to use data mining of patient information databases to look for patterns missed by traditional analysis. Researchers in the United Kingdom recently proposed to use data from hundreds of National Health Service wards for such data mining. The data analyzed by the researchers includes the number of infected patients on a ward, the type of treatments they received, and the hygiene and quarantine rules used by hospital staff to avoid or control infections. However, Mellmann and colleagues raise concerns, saying that although these data-mining “discovery” models are independent of an underlying hypothesis, they are usually less sensitive and specific, and once the unusual becomes usual it is no longer detected. Nonetheless, what is clear is that the days of infection outbreaks being detected solely by vigilant infectious-disease clinicians are over.

